# Recent Advances in the Treatment of Pulmonary Arterial Hypertension Associated with Connective Tissue Diseases

**DOI:** 10.3390/ph16091252

**Published:** 2023-09-05

**Authors:** Anna Smukowska-Gorynia, Weronika Gościniak, Patrycja Woźniak, Sylwia Iwańczyk, Karolina Jaxa-Kwiatkowska, Sylwia Sławek-Szmyt, Magdalena Janus, Jerzy Paluszkiewicz, Tatiana Mularek-Kubzdela

**Affiliations:** 1st Department of Cardiology, Poznan University of Medical Sciences, Długa 1/2 Street, 61-848 Poznan, Poland; weronika.gosciniak@skpp.edu.pl (W.G.); patrycja.wozniak@skpp.edu.pl (P.W.); sylwia.iwanczyk@skpp.edu.pl (S.I.); karolina.jaxa-kwiatkowska@skpp.edu.pl (K.J.-K.); sylwia.slawek@skpp.edu.pl (S.S.-S.); magdalena.janus@skpp.edu.pl (M.J.); jerzy.paluszkiewicz@skpp.edu.pl (J.P.); tatiana.mularek-kubzdela@skpp.edu.pl (T.M.-K.)

**Keywords:** PAH CTD, novel drugs, therapy, treatment, prognosis

## Abstract

Pulmonary hypertension (PH) is a severe vascular complication of connective tissue diseases (CTD). Patients with CTD may develop PH belonging to diverse groups: (1) pulmonary arterial hypertension (PAH), (2) PH due to left heart disease, (3) secondary PH due to lung disease and/or hypoxia and (4) chronic thromboembolic pulmonary hypertension (CTEPH). PAH most often develops in systemic scleroderma (SSc), mostly in its limited variant. PAH-CTD is a progressive disease characterized by poor prognosis. Therefore, early diagnosis should be established. A specific treatment for PAH-CTD is currently available and recommended: prostacyclin derivative (treprostinil, epoprostenol, iloprost, selexipag), nitric oxide and natriuretic pathway: stimulators of soluble guanylate cyclase (sGC: riociguat) and phosphodiesterase-five inhibitors (PDE5i: sildenafil, tadalafil), endothelin receptor antagonists (ERA: bosentan, macitentan, ambrisentan). Moreover, novel drugs, e.g., sotatercept, have been intensively investigated in clinical trials. We aim to review the literature on recent advances in the treatment strategy and prognosis of patients with PAH-CTD. In this manuscript, we discuss the mechanism of action of PAH-specific drugs and new agents and the latest research conducted on PAH-CTD patients.

## 1. Introduction

**Figure 1:**
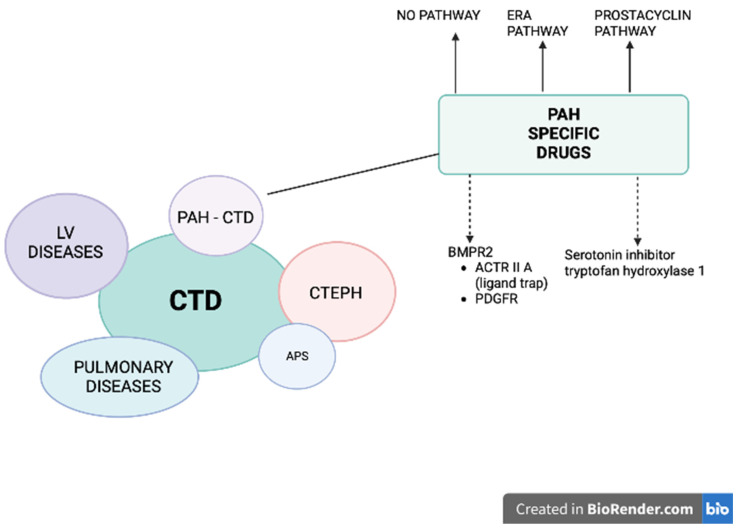
Patients with connective tissue diseases (CTD) may develop pulmonary hypertension (PH) belonging to different groups: (1) pulmonary arterial hypertension (PAH), (2) PH due to left heart disease, (3) PH secondary to lung disease and/or hypoxia (CTD patients mostly develop interstitial lung disease), and (4) chronic thromboembolic pulmonary hypertension (CTEPH), especially in the setting of antiphospholipid syndrome (APS). A specific treatment for PAH-CTD is currently available and recommended (solid arrows): prostacyclin derivative (treprostinil, epoprostenol, iloprost, selexipag), nitric oxide and natriuretic pathway: stimulators of soluble guanylate cyclase (sGC: riociguat) and phosphodiesterase-5 inhibitors (PDE5i: sildenafil, tadalafil) and endothelin receptor antagonists (ERA: bosentan, macitentan, ambrisentan). Two other pathways are under intensive investigation (dashed arrows): (1) affecting BMPR2: trapping the ligands of TGF-β and reducing the activity of ACTRIIA (sotatercept, KER-012) or inhibiting PDGFR (imatinib, seralutinib), (2) inhibiting the serotonin pathway by blocking the serotonin-producing enzyme tryptophan hydroxylase 1 (rodatristat ethyl).

Pulmonary hypertension (PH) is a vascular complication of connective tissue diseases (CTD), where, in addition to the symptoms of CTD with the involvement of many systems and organs on an autoimmune basis, pulmonary pressure is increased, and which, if left untreated, leads to severe right ventricular failure and death. Moreover, patients with CTD may develop PH belonging to different groups: (i) pulmonary arterial hypertension (PAH), (ii) PH due to left heart disease, (iii) PH secondary to lung disease and/or hypoxia and (iv) chronic thromboembolic pulmonary hypertension (CTEPH), especially in the setting of antiphospholipid syndrome (Figure 1) [1]. Pre-capillary PH is diagnosed by right-sided cardiac catheterization (RHC) when the following hemodynamic criteria are met: mean pulmonary arterial pressure (mPAP) > 20 mmHg, pulmonary vascular resistance (PVR) > 2.0 Woods units and pre-capillary wedge pressure (PCWP) ≤ 15 mmHg. PAH most often develops in systemic scleroderma (SSc), mainly in its limited variant [2,3,4]. The prevalence of pre-capillary PH (PAH and PH secondary to lung disease) in a large group of patients with SSc is estimated at 5–19% [2,3]. Other CTD associated with PAH development include systemic lupus erythematosus (SLE) [4,5,6], mixed CTD [4], rheumatoid arthritis [7], dermatomyositis [8] and Sjögren’s syndrome [9]. A specific treatment for PAH-CTD is currently available and recommended: prostacyclin derivative (treprostinil, epoprostenol, iloprost, selexipag), nitric oxide and natriuretic pathway: stimulators of soluble guanylate cyclase (sGC: riociguat) and phosphodiesterase-five inhibitors (PDE5i: sildenafil, tadalafil), endothelin receptor antagonists (ERA: bosentan, macitentan, ambrisentan) [10]. Until now, it was thought that the treatment of arterial pulmonary hypertension in patients with PAH-CTD is less effective than in patients without CTD [11,12,13]. However, the latest data indicate that implementing PAH-CTD treatment at the initial stage of the disease (World Health Organization [WHO] functional class [FC] I or II) is associated with a better prognosis [14,15]. Thus, we present a review of the literature on the treatment and prognosis of patients with PAH-CTD.

## 2. Diagnosis and Risk Assessment

It has been proven that asymptomatic patients with SSc should undergo screening to diagnose PAH as soon as possible and initiate specific treatment [16]. In 2011, Humbert et al. showed that patients with SSc undergoing screening with echocardiography compared to routine clinical practice are significantly more often diagnosed at an early stage of PAH in WHO FC I and II. It is now believed that all patients with SSc should undergo extended screening, preferably using a validated DETECT scale. This complex scale is characterized by higher specificity compared to echocardiography alone, leading to a faster diagnosis of PAH [17]. The DETECT scale includes forced vital capacity (FVC) percent predictive value, diffusion lung capacity for carbon monoxide (DLCO) percent predictive value, telangiectasias, anti-centromere antibody, N-terminal brain natriuretic propeptide (NT-proBNP), serum urate and right axis deviation on ECG. The next diagnostic step, according to the DETECT scale, is echocardiography, and the last is right-sided cardiac catheterization (RHC) [2]. The meta-analysis of Brown et al. confirmed improved survival in patients with SSc-PAH diagnosed as a result of screening [18]. Thus, the 2022 European Society of Cardiology (ESC)/European Respiratory Society (ERS) guidelines recommend annual evaluation of patients with SSc, preferably using the DETECT scale, and performing RHC for patients with breathlessness in whom non-invasive studies are unclear [10].

Death risk assessment is performed regularly during diagnosis and follow-ups every four to six months. According to the ESC guidelines, initial risk evaluation should include clinical, laboratory and echocardiographic evaluation, exercise capacity (six-minute walking test [6MWT]), heart pulmonary exercise test), hemodynamic parameters derived from RHC, as well as magnetic resonance (MRI) measurements [10]. Based on these parameters, patients are initially divided on a three-strata scale into high risk, intermediate risk and low risk of death. During scheduled visits, the highest predictive value is 6MWT, NT-proBNP or BNP, and the WHO-FC. Based on these few parameters, patients are divided on a four-strata scale into high-risk, intermediate-high-risk, intermediate-low-risk and low-risk patients. Another validated risk score scale used worldwide for risk stratification of PAH patients is REVEAL 2.0 and its shorter version REVEAL Lite 2, based on which patients are classified as high, intermediate or low risk of one-year mortality [19]. In addition to the basic parameters recommended by the ESC guidelines (NT-proBNP, 6MWT, WHO FC), the REVEAL 2.0 includes PAH etiology (+1 point for PAH-CTD), male age > 60 years old, estimated glomerular filtration rate (eGFR), heart rate, systolic blood pressure, all-cause hospitalizations ≤ 6 months, pericardial effusion on echocardiogram, percent predicted DLCO, mRAP and PVR.

The latest data from the COMPERA [20] and French registries [21] confirmed the highest mortality in the long-term observation of patients with PAH-CTD at high risk. In the Australian prospective study, independent predictive mortality factors in PAH-CTD patients were of older age, higher mPAP at PAH diagnosis, worse WHO FC and digital ulcers. Interestingly, the 6MWD was not predictive of mortality [22]. It can be explained by the observation that patients with PAH-CTD often have limited physical activity resulting from musculoskeletal involvement. Undoubtedly, the presence and severity of interstitial lung disease (ILD) significantly worsens the prognosis of patients with SSc. Moreover, sub-analysis has shown that patients with pre-capillary PH and extensive ILD have a worse prognosis compared to the subgroup of patients with mild ILD or without the ILD [12,23]. Furthermore, DLCO < 45% of the predicted value in the presence of other pulmonary functional tests within normal range can be diagnosed in PAH associated with SSc [24]. Severely reduced DLCO is related to a poor prognosis in different conditions [25]. However, the optimal treatment strategy in patients with PAH-CTD and severely reduced DLCO needs further investigation.

## 3. Therapy

Starting therapy at an early stage of PAH significantly improves the patients’ prognosis. In the French registry, three-year survival in patients with SSc was 80% when treatment was initiated in the NYHA (New York Heart Association)/WHO functional class II, 72% in stage III and 30% in IV [14].

The treatment of PAH-CTD involves drugs affecting several pathways/mechanisms, and the initial therapy depends on the severity of the disease. According to the ESC guidelines, like patients with idiopathic PAH, patients with PAH-CTD in the high-risk group should start PAH pharmacotherapy with a combination of triple therapy containing ERA, PDE5i and parenteral (s.c., i.v., inhaled) prostacyclin [10]. Patients with low- or intermediate-risk should start with dual combination therapy containing ERA, preferred macitentan or ambrisentan and PDE5i or riociguat. An initially implemented triple-combination treatment approach, including i.v./s.c. prostanoids should also be considered in intermediate risk with severe hemodynamic impairment, e.g., mean right atrial pressure (mRAP) ≥ 20 mmHg, cardiac index (CI) < 2.0 L/min/m^2^, stroke volume index < 31 mL/m^2^, PVR ≥ 12 Woods units. As comorbidities are common in PAH-CTD, the initial treatment of patients with cardiopulmonary risk factors (advanced age, obesity, atrial fibrillation, history of hypertension, coronary artery disease and/or low DLCO) should be considered as monotherapy with PDE5i or ERA.

The treatment, response to the therapy and management of patients with PAH-CTD should be provided by referral centers.

Furthermore, when triple therapy including parenteral prostacyclin analogue is initiated, it is recommended to refer these patients to specialized surgical centers for eligibility for bilateral lung transplantation. Lung transplantation is not contraindicated in patients with CTD-PAH. However, evaluating other potentially involved organs, especially the digestive system, kidneys, heart and skin, must be considered [15,26].

Another interventional treatment in advanced PAH includes balloon atrial septostomy (BAS) [27] by performing an interatrial shunt, leading to decompression of the right atrium and ventricle and increased systemic blood flow at the expense of arterial oxygen saturation. BAS should be performed in the experienced PAH centers as a bridge to lung transplantation or ultimate therapy, preferably with atrial flow regulator (AFR) [28]. Other interventional treatments are under clinical investigation: denervation of pulmonary arteries (PADN) by using the radiofrequency ablation [29] or intravascular ultrasound catheter [30]. The latter was characterized by good safety measures and showed improvement in 6MWT and reduced PVR.

Analysis of subgroups of patients with PAH-CTD and PAH-SSc from randomized trials with sildenafil, tadalafil, bosentan, ambrisentan, macitentan, treprostinil, iloprost, riociguat and selexipag showed a beneficial effect. These subsections present alternative pharmacological targets and their efficacy in CTD patients with PAH.

### 3.1. Prostacyclin Pathway

Prostacyclin analogs bind to the prostacyclin receptor, leading to increased cyclic adenosine monophosphate (cAMP), resulting in vasodilation, antithrombotic effects and cytoprotective and anti-proliferative activities effects. Four agents have been licensed by the Food and Drug Administration (FDA) and/or European Medicines Agency (EMA) for the treatment of PAH: epoprostenol (iv), treprostinil (i.v., s.c., inhaled, oral), iloprost (inhaled) and selexipag, an orally active, selective prostacyclin receptor agonist. The most frequently reported adverse events observed with the aforementioned drugs are due to systemic vasodilation and include headache, flushing, jaw pain and diarrhea.

#### 3.1.1. Epoprostenol

Epoprostenol needs a continuous i.v. infusion pump and permanent tunneled catheter as its half-life is estimated to be three to five minutes. Intravenous epoprostenol is considered the first-line agent in high-risk PAH. In a randomized trial involving 111 patients with SSc spectrum, three months of intravenous epoprostenol infusion improved physical performance, hemodynamic parameters and the WHO FC [31]. The delivery system is associated with several main complications including focal system infection, catheter obstruction and sepsis.

#### 3.1.2. Iloprost

Iloprost is a prostacyclin analog, which is delivered by direct drug inhalation. It was investigated in one RCT, in which six to nine iloprost inhalations were compared with placebo in treatment-naive patients with either PAH or CTEPH [32]. The trial included patients with severe forms of PAH-CTD. Inhaled iloprost was reported as significant in terms of exercise capacity, functional class and quality of life improvement, along with PVR, and clinical adverse events decreased in patients administered iloprost compared with the placebo group. 

#### 3.1.3. Treprostinil

Treprostinil is available for s.c., i.v., inhaled or oral administration (however, the availability of the form of the drug and the route of its administration depends on the country). In patients with PAH-CTD, treprostinil s.c. improved exercise capacity (6MWT), hemodynamics (significantly decreased PVR index) and symptoms [33]. The most common adverse effects included infusion-site pain. It led to treatment discontinuation in 8% of cases [34]. Treprostinil may be administered via implantable i.v. pumps due to its chemical stability. This possibility substantially improved the adherence and decreased the occurrence of line infections. 

#### 3.1.4. Selexipag

Selexipag is a selective prostacyclin receptor agonist, chemically distinct from prostacyclin, which is administered p.o. The GRIPHON study [35], which enrolled 1156 patients, revealed that selexipag alone or in addition to mono- or dual therapy with an ERA and/or a PDE5i reduced the risk of morbidity/mortality events by 40%. Results from the GRIPHON study also showed that selexipag’s long-term safety and tolerability profile was in accordance with previously published data over shorter periods. A sub analysis of the 334 PAH-CTD patients [36] confirmed that selexipag reduced the risk of the primary composite endpoint of morbidity/mortality by 41% versus placebo (HR 0.59; 95% CI 0.41–0.85). The effect was consistent in patients with PAH-CTD irrespective of PAH therapy at baseline. The risk reduction conferred by selexipag versus placebo was higher in patients with PAH-SSc than in those with SLE-PAH (44% and 34%, respectively), emphasizing the crucial role of the prostacyclin pathway in SSc. The patients reported the side effects as headache, diarrhea, nausea and jaw pain. 

### 3.2. Endothelin Receptor Antagonists’ Pathway

#### 3.2.1. Ambrisentan

Ambrisentan, an oral ERA, blocks the endothelin A receptors. The doses in adults are 5 mg and 10 mg in a single dose. ARIES 1 and 2 assessed the efficacy and safety of ambrisentan in PAH [37]. An increased incidence of peripheral edema was reported with ambrisentan use, while no evidence reported aminotransferase concentrations greater than three times the upper normal range limit. The ARIES-E clinical trial subgroup analysis evaluated 124 patients with PAH-CTD, among which 62.6%, 57.3% and 58.2% of PAH-CTD patients treated with ambrisentan exhibited increased 6MWD at one, two and three years, respectively. At three years, 64% of patients were free from clinical worsening and 76% were still alive [38].

#### 3.2.2. Bosentan

Bosentan is an oral, dual ERA that improves exercise capacity. Along with the increase in its dose, the results showed an increase in liver transaminases in approximately ten percent of investigated patients (reversible after dose reduction or discontinuation). In patients receiving bosentan the aminotransferase level should be assessed monthly. In the 351 and BREATHE-1 studies, the efficacy of bosentan versus placebo showed changes in exercise capacity (6MWT) [39,40]. In patients with PAH-CTD (n = 66) at the end of the study, the 44 patients treated with bosentan did not present a worsening of 6MWT, unlike the 22 patients on placebo. There was no significant difference in the PAH-CTD patients, contrasting with the overall study population [41]. The lack of improvement in 6MWT with bosentan, specifically in these patients, may be caused by increasing musculoskeletal dysfunction [42]. Nevertheless, in the EARLY study, which evaluated the effectiveness of bosentan in mildly symptomatic (WHO FC II) patients, regardless of PAH etiology, in the bosentan group, 6MWT results improved from baseline (11.2 m, 95% CI −4.6 to 27.0) while in the placebo group, compared to the 6MWT result (−7.9 m, −24.3 to 8.5), confirming a mean treatment effect of 19.1 m [43]. Furthermore, in the sub analysis of the long-term EARLY extension study, survival rates among the 64 PAH-CTD patients, who subsequently received bosentan, were 86% and 73% at one and two years, respectively. Long-term results from this study also showed elevations in aminotransferase levels above three times the upper limit in 16.8% over a median exposure of 51 months. Most abnormalities associated with bosentan occurred during the first six months of treatment (11.6%) and were reversible after drug discontinuation [44].

#### 3.2.3. Macitentan

Macitentan is an oral, dual ERA that increases exercise capacity and reduces a composite endpoint of clinical worsening in patients with PAH. The SERAPHIN trial [45] confirms the long-term efficacy of macitentan (dual ERA) in doses of 3 or 10 mg p.o. Pulido et al. observed increased exercise capacity and reduced composite endpoint of clinical worsening in patients with PAH. The subgroup analysis of PAH-CTD (n = 224) showed likely clinical benefit. Macitentan significantly reduced morbidity and mortality (45%) in all patients vs. placebo. The effect of macitentan was also maintained in patients on background-specific therapy. No liver toxicity has been shown. Anemia with hemoglobin level ≤ 8 g/dL was reported in 4.3% of patients receiving 10 mg of macitentan (probably because of fluid retention and hemodilution). 

### 3.3. Nitric Oxide Pathway

Reduced nitric oxide (NO) availability is related to PAH. NO induces vasodilation and inhibits vascular proliferation by increasing cyclic guanosine monophosphate (cGMP) production. Phosphodiesterases (PDEs) are enzymes that inactivate cGMP. Phosphodiesterase 5 inhibitors (PDE5-I) (sildenafil and tadalafil) slow the breakdown of cGMP, and the soluble guanylate cyclase agonist (riociguat) stimulates cGMP production. 

Soluble guanylate cyclase (sGC) stimulation by nitric oxide produces the intracellular second messenger cyclic guanosine monophosphate (cGMP). A negative feedback loop controls this pathway via the cGMP degradation of by the different PDEs. The subtype 5 (PDE5) is abundantly expressed in the pulmonary vasculature. PDE-5 inhibitors and sGC stimulators must not be combined with each other nor with nitrates, as this can result in systemic hypotension. 

#### 3.3.1. Sildenafil

Sildenafil is an orally active, selective inhibitor of type five cGMP phosphodiesterase (PDE5i). The SUPER-1 study showed a significant improvement of the 6MWT from baseline to week 12 (primary endpoint) in patients treated with sildenafil (20, 40 or 80 mg three times daily) vs. placebo [46]. In the subgroup of CTD-PAH patients, a significant improvement in mPAP and PVR was observed in patients treated with sildenafil vs. the placebo group. However, the effect on 6MWT was less evident than in the entire SUPER-1 study population. Sildenafil was generally well tolerated. Most side effects reported were mild to moderate and were mainly related to vasodilation (headache, flushing and epistaxis).

#### 3.3.2. Tadalafil

Tadalafil is a once-daily administered PDE5i. In the PHIRST study, patients treated with tadalafil (in dose of 2.5 mg, 10 mg, 20 mg or 40 mg) showed a significant improvement of the 6MWT at week 16 from baseline compared to placebo, along with a significant decrease in mPAP. PAH-CTD subgroup (n = 79) showed improvement of 6MWT in all doses: 2.5 mg, 10 mg, 20 mg and 40 mg daily; however, as in the entire group, the greatest clinical improvement was achieved in patients treated with 40 mg. Moreover, greater improvement was observed in the CTD-PAH group compared to the group of another etiology of PAH. The side effects were similar to that of sildenafil [47].

#### 3.3.3. Riociguat

As mentioned before, the PDE5is affect the nitric oxide–cGMP pathway by slowing cGMP degradation. Meanwhile, the sGCs improve the production of cGMP by directly stimulating the enzyme, regardless of the presence and the absence of endogenous nitric oxide. The PATENT-1 study [48] evaluated the efficacy of riociguat treated up to 2.5 mg three times daily vs. placebo. Riociguat was well tolerated in the entire PAH population. Riociguat positively affected the endpoints in patients with PAH-CTD, including 6MWT, PVR and CI, though improvements were less pronounced compared to the overall PATENT-1 population. Patients pretreated with ERAs (53%) manifested improvement in 6MWT, supporting the potential use of riociguat in combination therapy in this subgroup. Of note, the two-year survival rate in the long-term extension study PATIENT-2 was comparable in patients with PAH-CTD and IPAH (93%). The side effects were similar to other drugs of the PDE5is group. 

### 3.4. Novel Drugs

#### 3.4.1. Sotatercept

**Figure 2:**
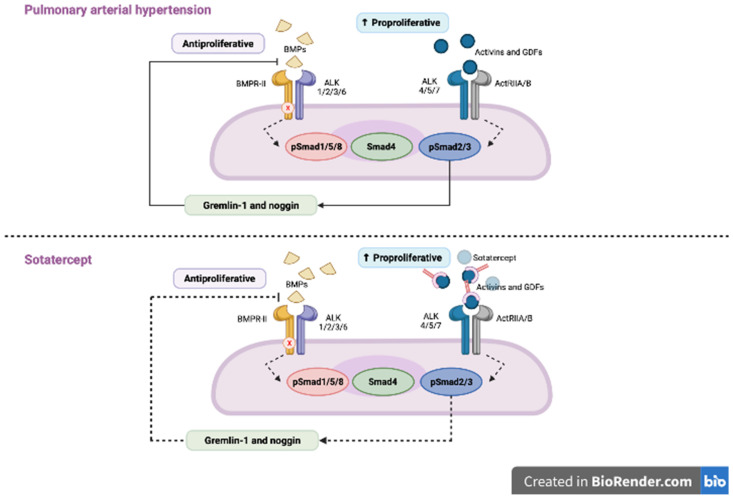
In patients with pulmonary arterial hypertension (PAH) BMP signaling is impaired, which leads to the downregulation of anti-proliferative SMAD 1/5/9 and upregulation of pro-proliferative SMAD 2/3 and, in turn, to the proliferation of the smooth muscle cells in pulmonary arterioles. Sotatercept, by trapping the ligands of TGF-β family and reducing the ACTRIIA activity, restores the balance between SMAD2/3 and SMAD 1/5/9. Adapted from Ref. [50].

BMPR2 mutations, the transforming growth factor-β (TGF-β) superfamily, are crucial in heritable PAH and acquired PAH [49]. Thus, sotatercept a recombinant fusion protein consisting of the extracellular part of the human activin receptor type IIA, which is linked to the Fc piece of human IgG1 restores balance between growth-promoting and growth-inhibiting signaling. This fusion protein traps activins and growth differentiation factors (by reducing ACTRIIA-Smad2/3 signaling) involved in PAH [50]. The mechanism of action of sotatercept is presented in Figure 2. The STELLAR trial was a randomized double blind trial investigating sotatercept on top of double or triple PAH-specific therapy vs. placebo (n = 163 in the sotatercept group and n = 160 in the placebo group and among them 17.8% were patients with PAH-CTD). Sotatercept was administered by subcutaneous injections every 21 days in an initial dose of 0.3 mg/kg. The dose was increased to 0.7 mg/kg on the second visit. According to the results of this trial, patients in the sotatercept group had significantly improved exercise capacity and reduced risk of clinical worsening or death by 84% compared with placebo for a hazard ratio (HR) of 0.16 (95% CI, 0.08–0.35; *p* < 001). The most common adverse events of special interest were bleeding events (21.5%, mostly nonserious epistaxis and gingival bleeding), telangiectasia (10.4%) thrombocytopenia (6.1%), increased hemoglobin level (5.5%) and increased blood pressure (3.7%) [51]. Patients who completed the STELLAR trial can be enrolled into the ongoing SOTERIA trial (NCT04796337). The impressive results of the STELLAR trial provided promising data for novel therapeutic strategies for PAH. It might be a novel approach to the treatment of PAH in combination with already approved therapies. 

#### 3.4.2. Tocilizumab

In the opposite of sotatercept, tocilizumab (a cytokine IL-6 inhibitor) did not show any consistent treatment effect, including a change in pulmonary vascular resistance. Moreover, inflammatory markers had no predictive value in terms of response to treatment. Mendelian randomization did not confirm an effect of the lead *IL6R* variant on the risk of PAH (OR 0.99, *p* = 0.88) [52]. 

#### 3.4.3. Rituximab

Rituximab (a monoclonal antibody to CD20 which is associated with B-cell depletion) in the SSc-PAH population showed insignificant change in 6MWD at 24 weeks, which favored this treatment approach [53]. However, the rituximab treatment was safe and well tolerated.

### 3.5. Ongoing Trials in PAH Population

There are several ongoing clinical trials for novel PAH therapies. However, only one study on the effects of oral **ifetroban** (NCT02682511) exclusively evaluates patients with SSc-associated PAH. Oral ifetroban is a potent and selective thromboxane (TX) receptor antagonist. Previously, it was shown that TX is associated with hypoxia-induced pulmonary hypertension through platelet activation and inhibition of angiogenesis [54,55]. In a rat model of PAH, ifetroban partly reversed the platelet activation and deposition induced by hypoxia [56]. In addition, in a mice model of PAH with knockout of cyclooxygenase 2 (COX 2), ifetroban inhibited the effect of deleting the COX-2 gene, diminishing the hypoxia-induced rise in right ventricle end systolic pressure and intravascular thrombosis [57].

The IMPAHCT trial (NCT05036135) is a Phase 2b/Phase 3 study to evaluate the safety and efficacy of AV-101 (dry powder **inhaled imatinib**) in patients with PAH on at least double-drug background therapy. Imatinib is an antiproliferative drug invented to target the BCR-ABL tyrosine kinase in patients with chronic myeloid leukemia and widely used in oncology. The literature contains evidence from animal models and human disease suggesting that platelet-derived growth factor (PDGF) and stem cell factor receptor (c-KIT) pathways play a crucial role in vascular smooth muscle cell hyperplasia and proliferation. Moreover, the inhibitory activity of imatinib on PDGF receptors α/β and c-KIT indicate that it may be effective in PAH [58,59]. Previously, in the IMPRES trial, oral imatinib in a dose of 400 mg worked on top of dual or triple PAH-specific therapy. Patients in the oral imatinib group had significant improvement in 6MWT (+32 m) and in hemodynamics (reduction in PVR of 32%, increase in CO of 0.9 L/min and reduction in mPAP of 5.2 mmHg). However, the main limitation in the further investigation of oral imatinib was the poor tolerability, including serious adverse bleeding events, such as subdural hematoma [60]. Thus, inhaled imatinib delivered by a dry powder oral inhaler designed to deliver the drug directly to the disease organ and limit systemic exposure has a much better safety profile.

**Figure 3:**
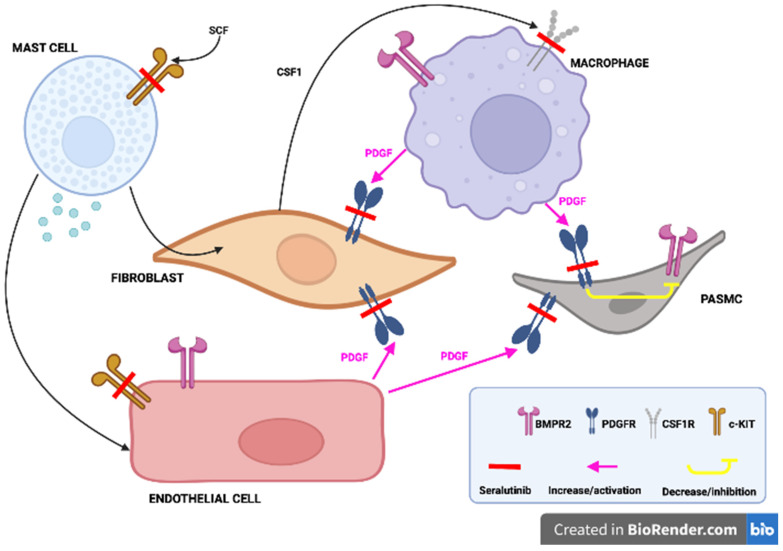
Seralutinib, by blocking the PDGF receptors located on the fibroblasts and pulmonary arterioles smooth muscle cells (PASMC), promotes the BMPR2 function, in addition, sotatercept, by inhibiting the c-KIT receptors located in the mast cells and endothelial cells as well as CSF-1 receptors located in the macrophages, favors the BMPR2 function and decreases inflammation. Adapted from Ref. [61].

**Seralutinib** is another orally inhaled kinase inhibitor (inhibitor of PDGFR/CSF1R/c-KIT) which inhibits lung PDGFRα/β phosphorylation and induces lung BMPR2 protein expression. An ongoing open-label extension study (NCT04816604) will evaluate the long-term effects of seralutinib in patients who previously were enrolled in a GB002 PAH trial. In the study of Garkin et al. inhaled seralutinib was examined in two models of PAH in rats. According to the study results, seralutinib improved hemodynamic parameters, reduced NT-proBNP, reversed remodeling of pulmonary vascular arteries and improvement in inflammatory biomarkers. In addition, seralutinib was characterized by higher efficacy compared to imatinib [61]. The mechanism of action of seralutinib (which is similar to imatinib) and its potential role in PAH treatment is presented in Figure 3.

**Figure 4:**
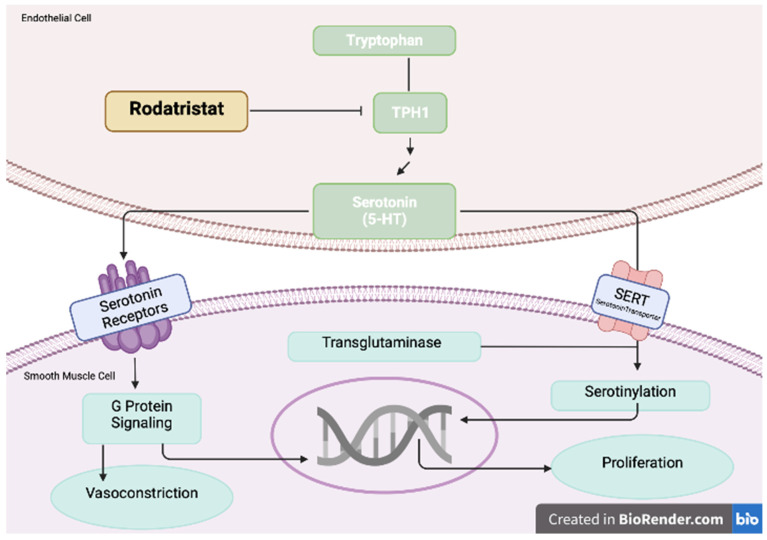
Increased levels of serotonin promote pulmonary arterial smooth muscle cell proliferation and contraction. Rodatristat ethyl blocks the serotonin-producing enzyme tryptophan hydroxylase 1 (TPH1). Adapted from Ref. [62].

The ELEVATE 2 is a double-blind trial with **rodatristat ethyl**, which blocks the serotonin-producing enzyme tryptophan hydroxylase (TPH, NCT04712669). Increased levels of serotonin promote pulmonary arterial smooth muscle cell proliferation and contraction which is a hallmark of PAH development. Rodatristat ethyl is a first-in-class orally administered prodrug, which does not cross the blood–brain barrier and is able to exclusively reduce peripheral and lung serotonin. By decreasing the concentration of circulating serotonin, rodatristat ethyl is a promising novel agent in PAH treatment [62]. The mechanism of rodatristat ethyl, by inhibiting the serotonin pathway, is shown in Figure 4.

The ROR-PH-301 Phase III study investigates **ralinepag**—a novel, oral, selective prostacyclin receptor agonist administered once daily, with optimized pharmacokinetics (NCT03626688). The long-term data from the ralinepag Phase II open-label extension study (n = 41, among them 35.6% were patients with PAH-CTD) showed a mean decrease of 52.2 dyn·s/cm^5^ of PVR and a mean increase of 52.4 m in 6MWT. The most common adverse events were similar to those known to be related to prostacyclin receptor agonist therapy: headache, diarrhea and nausea [63].

The approach 2 trial focused on **apabetalone**, which is a selective inhibitor of the Bromodomain-containing protein 4 (BRD4) involved in epigenetic regulators of gene expression. The authors assessed the efficacy of a BRD4 inhibitor compared to a placebo in adult subjects with PAH on stable background therapy (NCT04915300). In the study of Van der Feen et al., oral apabetalone (RVX208) restored to a normal phenotype smooth muscle cells and microvascular endothelial cells isolated from distal pulmonary arteries of PAH patients. Moreover, it reversed vascular remodeling in two PAH models in rats [64].

The SATISFY-JP Trial (NCT05679570) is a phase 2 study with **satralizumab**, which is an anti-IL-6 receptor antibody [65]. 

**GMA301** is a humanized monoclonal antibody against endothelin receptor A. It was investigated in a randomized, double-blind, placebo-controlled, dose escalation study in patients with PAH (NCT04503733) [66]. 

The VIPAH-PRN 2B is a phase 2b, open-label, single dose study that aimed to evaluate the safety and efficacy of RT234 (**inhaled vardenafil**) on exercise parameters assessed by cardiopulmonary exercise testing (CPET, NCT04266197) [67]. 

The INSIGNIA-PAH is a phase 2/phase 3 study that aimed to assess the efficacy and safety of an **inhaled sGC stimulator** (**MK-5475-007**) in PAH (NCT04732221). In a Phase 1 study, treatment with MK-5475 revealed reductions in pulmonary vascular resistance and was well tolerated [68].

**Olaparib** is a DNA damage and poly-[ADP-ribose] polymerases [PARP] inhibitor registered in oncological treatment [69]. It is currently being investigated in PAH patients (NCT03782818).

Last, but not least, KER-012-A201 is a randomized, Phase 2, double-blind, placebo-controlled study designed to investigate the safety and efficacy of **KER-012** in combination with background therapy (NCT05975905). The first patients are planned to be recruited in 2023. KER-012 is a recombinant fusion protein consisting of a modified ligand-binding domain of the TGF-β receptor (activin receptor type IIA) that is fused to a part of the human antibody (Fc domain). KER-012 is administered subcutaneously and its mechanism of action is similar to the sotatercept: KER-012 traps select TGF-β superfamily ligands and in turn inhibits the pro-proliferative SMAD 2/3 and favors antiproliferative SMAD1/5/9 (promotes BMP signaling). Thus, this agent can potentially reverse the vascular remodeling in PAH patients.

## 4. Summary

Some of the studies investigating PAH-specific drugs have shown that the effect of treatment was worse in patients with CTD-PAH than in patients with idiopathic PAH [70]. However, the primary endpoint in these studies was most often the 6MWT. Nevertheless, the results of a 2021 meta-analysis of 11 randomized trials (n-4329, n-1267 CTD-PAH) and 19 registries (n-9739, n-4008 CTD-PAH) with at least 30-person subgroups with CTD-PAH from 2000–2019 showed a beneficial effect of modern PAH-specific treatment in both CTD-PAH and PAH groups. The meta-analysis of randomized trials in which the endpoint was composite (time to event mortality/morbidity including death, worsening, hospitalization for prostacyclin, lung transplantation or septostomy) showed the same 36% reduction in the risk of incident mortality/morbidity in patients treated with PAH-specific therapy in comparison with placebo in both the overall group and the subgroup with CTD-PAH. However, at baseline, both in the RCTs and in the registries, patients with CTD-PAH had a lower mean 6MWT and were older compared to the overall PAH population. In addition, PAH-CTD patients had a higher mortality rate when compared with other groups of patients with PAH (three-year survival was 62% in patients with CTD-PAH and 72% in all patients) [66]. Nevertheless, the three-year survival of patients with CTD-PAH after 2010 was better than before 2010 (73% vs. 65%, respectively) [71]. The AMBITION study (whose primary endpoint was clinical worsening) showed that in all patients, regardless of PAH etiology, initial composite treatment with tadalafil and bosentan was preferable to initial monotherapy. These results were proved in a post hoc analysis exclusively assessing patients with PAH-CTD (187 patients with PAH-CTD, of whom 118 had SSc-PAH) [72].

## 5. Conclusions

PAH-CTD is a progressive disease that must be detected early (patients with SSc should be screened annually), and composite drug therapy is recommended to be implemented as early as possible [73]. Despite a serious prognosis, PAH-CTD can be effectively treated with PAH-specific drugs. The number of drugs (monotherapy, dual or triple therapy) initiated in PAH-CTD depends on the risk stratification and presence of comorbidities at the time of the diagnosis. There are several intensively investigated novel drugs, from which the most promising are agents affecting BMPR2 signaling (sotatercept), PDGFR (imatinib, seralutinib) and the tryptophan pathway (rodatristat ethyl).

## Data Availability

Data sharing is not applicable.
